# Omnipresent distribution of herbicides and their transformation products in all water body types of an agricultural landscape in the North German Lowland

**DOI:** 10.1007/s11356-021-13626-x

**Published:** 2021-04-13

**Authors:** Uta Ulrich, Matthias Pfannerstill, Guido Ostendorp, Nicola Fohrer

**Affiliations:** 1 Institute of Natural Resource Conservation, Christian-Albrechts University Kiel, Olshausenstr. 75, 24118 Kiel, Germany; 2 State Agency for Agriculture, the Environment and Rural Areas Schleswig-Holstein, Hamburger Chaussee 25, 24220 Flintbek, Germany; 3 Department of Environmental Health, State Social Services Agency Schleswig-Holstein, Brunswiker Straße 4, 24105 Kiel, Germany

**Keywords:** Flufenacet, Metazachlor, Drainage, Shallow groundwater, Drinking water, Stream water, Rainfall pattern, Physical-chemical properties

## Abstract

The research of the environmental fate of pesticides has demonstrated that applied compounds are altered in their molecular structure over time and are distributed within the environment. To assess the risk for contamination by transformation products (TP) of the herbicides flufenacet and metazachlor, the following four water body types were sampled in a small-scale catchment of 50 km^2^ in 2015/2016: tile drainage water, stream water, shallow groundwater, and drinking water of private wells. The TP were omnipresent in every type of water body, more frequently and in concentrations up to 10 times higher than their parent compounds. Especially metazachlor sulfonic acid, metazachlor oxalic acid, and flufenacet oxalic acid were detected in almost every drainage and stream sample. The transformation process leads to more mobile and more persistent molecules resulting in higher detection frequencies and concentrations, which can even occur a year or more after the application of the parent compound. The vulnerability of shallow groundwater and private drinking water wells to leaching compounds is proved by numerous positives of metazachlor-TP with maximum concentrations of 0.7 μg L^−1^ (drinking water) and 20 μg L^−1^ (shallow groundwater) of metazachlor sulfonic acid. Rainfall events during the application period cause high discharge of the parent compound and lower release of TP. Later rainfall events lead to high displacement of TP. For an integrated risk assessment of water bodies, the environmental behavior of pesticide-TP has to be included into regular state-of-the-art water quality monitoring.

## Introduction

The environmental fate of pesticides and their effect on ecosystems and at the single species level have been investigated intensively in the last decades with various focusses such as hydrological conditions, physical-chemical properties of the compounds, or soil parameters (Wołejko et al. 2020). It has become apparent that pesticides can be present in the environment though the concentrations are below the limit of quantification (LOQ) or limit of detection (LOD) (Längin et al. 2008). Additionally, several biotic and abiotic processes have been identified which alter the chemical structure of molecules and transform them into so-called transformation products (TP) (Boxall et al. 2004; Fenner et al. 2009, 2013). This is not a specific behavior of pesticides but of chemical compounds in general. In the case of modern pesticides, rapid degradation to TP is intended to avoid persistence and bioaccumulation as is well known for chlorinated compounds. A general problem is the lack of knowledge about emerging TP and the insufficient data available concerning their ultimate fate in the environment (Dieter 2010). TP show different physical-chemical properties than their parent compounds (PC), especially in terms of mobility, persistence, and (eco)-toxicity. Belfroid et al. (1998), Boxall et al. (2004), and Kiefer et al. (2019) observed an increase of polarity leading to a decrease of the organic carbon adsorption coefficient (Koc) of the observed TP/PC combination by a factor of up to 10. Furthermore, TP tend to show a higher persistence than their PC (Boxall et al. 2004; Fenner et al. 2013; Lewis et al. 2016; Kiefer et al. 2019). The increase of mobility and persistence in turn causes higher detection frequencies and concentrations in samples of all environmental media (Ulrich et al. 2018; Willkommen et al. 2019; Kiefer et al. 2019). In terms of ecotoxicity, Sinclair and Boxall (2003) observed that 70% of the TP in their study showed a similar or lower ecotoxicity for fish, daphnids, or algae than their corresponding PC, but in 30%, the TP was more toxic.

The type of transformation depends on the compartment where the pesticide is located. In soils and sediments, the transformation by microorganisms is dominant. Certain species such as *Chlorella*, *Arthrobacter*, *Aspergillus*, *Pseudomonas*, and *Chlorella* are able to initiate or support chemical reactions like adsorption, hydrolysis of special functional groups, oxidations, and enzymatic mineralization (Fenner et al. 2013; Kumar et al. 2018). In air, in the upper layer of surface water and on the top soil, direct and indirect photolysis are the main transformation processes (Kiss and Virág 2009; Hensen et al. 2019). Hydrolysis can lead to the transformation of pesticides in surface and groundwater. Anaerobic conditions in all media lead to reducing processes which alter the PC (Fenner et al. 2013). Besides prevalent conditions in the specific compartments, the physical-chemical PC characteristics and environmental parameters such as pH, temperature, organic carbon, or oxygen contents proved to have relevant effects on transformation processes (Berkowitz et al. 2008; Farlin et al. 2013; Dechene et al. 2014; Marín-Benito et al. 2019).

So far, PC and their TP have been quantified in different water types including drinking water (Rodriguez-Mozaz et al. 2004; Reemtsma et al. 2013a), groundwater (Albanis et al. 1998; Bottoni et al. 2013; Farlin et al. 2017), surface water (Olsson et al. 2013; Reemtsma et al. 2013b; Masiá et al. 2015), and in soil (Marín-Benito et al. 2019; Silva et al. 2019; Willkommen et al. 2019). These observations in different compartments were carried out as singular sampling or in low temporal resolution and were isolated from each other. Hence, the temporal dynamics of TP findings, spatial distribution, or distribution in different media within one region/catchment have not been investigated so far. Therefore, the aim of our study was to close this gap by applying an integrated assessment approach for evaluating the prevalence of emerging pollutants such as TP and its distribution in different types of water within one catchment using high temporal resolution. It can be seen as a starting point of further process-oriented research on the environmental fate of TP. Our research questions to be answered are as follows:

1. Which water bodies are subject to inputs of TP? Do they show similar or distinct patterns?
2. How do distinct hydrological conditions affect the export of TP in fast-reacting compartments such as drainage water and stream water?
3. How vulnerable is groundwater to leaching of pesticides and their TP?

## Material and methods

Within our sampling campaign, groundwater from private drinking water wells and shallow groundwater monitoring wells including tile drainage water and stream water at the outlet of the catchment of the stream Kielstau were sampled in 2015 and 2016 using different temporal resolutions.

### Study site

**Fig. 1:**
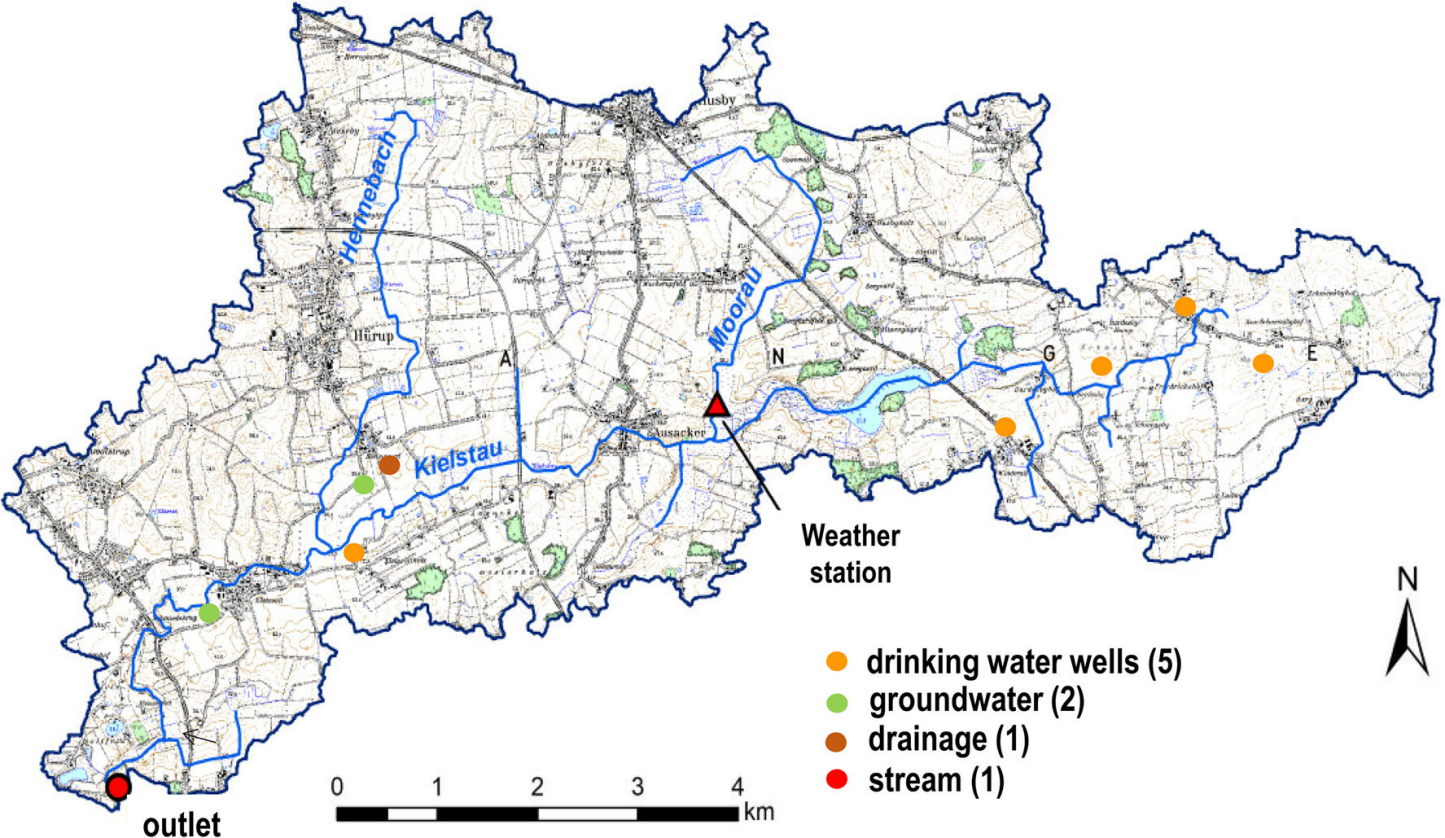
Catchment of the stream Kielstau with sampling points

The investigations were carried out in the catchment of the lowland stream Kielstau in the North of Germany. The catchment has a size of 50 km^2^ (Fig. 1). The catchment is characterized by low population (eight municipalities with scattered farms in between) and intensive agricultural use. The main crops are rape (*Brassica napus*), winter wheat (*Triticum aestivum*), winter barley (*Hordeum vulgare*), and corn (*Zea mays*). Dominant soils in the east and south are fertile brown soils and luvisols, while gley soils and groundwater-dependent peat soils dominate stream valleys and depressions. The slopes vary in the range of 0–10 % with a mean slope of 2%. Typical lowland characteristics such as shallow groundwater levels (Pfannerstill et al. 2014, 2015) and a high share of 38% drained agricultural area can be observed (Fohrer et al. 2007). The glacial period led to a high number of ponds located in the agricultural fields (Ulrich et al. 2018). The annual mean temperature is 8.5 °C, and the annual mean precipitation is 850 mm (30 years mean 1980–2010, Deutscher Wetterdienst DWD), with highest values from October to January. The Kielstau stream has a length of 17 km and is characterized according to the European Water Framework Directive as classification type 16 “gravel dominated lowland stream.” The stream is fed by various small tributaries, open drainage ditches, and drainage pipes and has been straightened artificially in several sections. Further detailed information is reported in Fohrer and Schmalz (2012) and Wagner et al. (2018).

### Sampling

#### Shallow groundwater

Two groundwater wells were sampled monthly for a period of 2 years (Table 1.) Sampling was carried out according to DIN EN ISO 5567–1:2007 – 04, DIN 38 402–13 (1985), DIN EN ISO 5667-3:2012, LAWA AQS-Merkblatt P-8/2 (1995-02), and DVGW W 112 (A):2011-10. A submersible pump (Grundfos MP1) was used to pump water out of the groundwater wells until physico-chemical parameters were constant (WTW 3540) and water around the filter stripe was exchanged three times. Water samples were filled into two glass bottles of 30 ml and were cooled during transport into the laboratory. The first groundwater well (GW1) was installed at a sandy site, whose hydraulic gradient is directed towards the Kielstau stream. From surface to 1.2 m below the surface, there was fine sand (0.063– - < 0.2 mm) and material following middle sand (0.2– < 0.63 mm) until the end of the borehole of 8 m. The filter strip was installed with surrounding filter sand (0.4–0.8 mm) between 3.5 and 7.5m below the surface. Above the filter strip, betonit was filled into the borehole around the pipe. The mean groundwater level within the well was 2.76 m below the surface. At the beginning of our sampling in September 2015, winter rapeseed was grown at the surrounding area of the well, and metazachlor was applied in autumn. The second groundwater well (GW2) was installed at a loamy site, which is located at the transition of a hillside and a depression, where the Kielstau flows through. The first 1.5-m underground are characterized by silt (0.063–0.02 mm). The filter strip of the well was installed in fine sand (0.063– < 0.2 mm) and was installed with surrounding filter sand (0.4–0.8 mm), which accordingly starts at 1.5m and ends at 4.5m below the surface. As for GW1, betonit was used to seal the borehole around the pipe. In the surrounding area, winter grain and winter rapeseed were grown alternately according to the common rotation of winter wheat-winter barley-winter rapeseed.

**Table 1:** Sampling periods and intervals during the monitoring periods 2015 and 2016

Type of water body	Sampling period	Sampling interval
Groundwater	01/09/2015–15/09/20117	Monthly
Drinking water	11/2015–09/2017	Quarterly
Drainage water	06/10–31/12/2015 25/8–06/12/2016	Daily
Stream water	25/8–31/12/2015 22/8–22/11/2016	Daily, starting with the beginning of the application period

#### Drinking water

The drinking water from households which are not connected to the public drinking water supply but receive drinking water from privately owned small-scale water supplies was sampled at five locations after extraction and before supply to the drinking water system. The samples were taken after 3 min of water flow and filled directly into 30 ml glass jars. DW 1 and 5 each provides for one household; DW 2 provides for three households, while DW 3 is counted as a small group of suppliers with a provision of <10 m^3^/day or for 50 people, and DW 4 provides approximately 50 m^3^/d for 400 people. Sampling intervals are listed in Table 1.

#### Tile drainage water

A drainage pipe which drains 4–8 ha of one field (30 ha) was sampled with an automatic sampler (ISCO 6712). Eight time-weighted samples per day were aggregated to a daily mixed sample of 350 ml (glass bottle). Sampling intervals are listed in Table 1.

#### Stream water

The stream water was sampled at the outlet of the catchment with an automatic sampler (ISCO 6712). Eight time-weighted samples per day were aggregated to a daily mixed sample of 350 ml (glass bottle). Sampling intervals are listed in Table 1.

#### Hydrological parameters

In the drainage pipe, the water level was measured in 15-min intervals with a pressure sensor (SEBA, dipper PT) and aggregated to daily mean values. Twice a week, discharge and water level were measured manually, and a rating curve was generated, so it was possible to calculate the discharge.

In the stream, the discharge at the outlet (official gauge of the Landesamt für Landwirtschaft, Umwelt und ländliche Räume, LLUR) was measured continuously with a pressure sensor (SEBA, dipper PT) by the LLUR (2017).

Precipitation was measured in 10-min intervals within the catchment with an unheated tipping bucket rain gauge (Campbell Scientific 52203). Values were aggregated to daily sums.

Groundwater levels were measured monthly at each sampling date with an electric contact gauge (Ott, Ott PLS).

### Selected target compounds

For this study, the herbicides metazachlor and flufenacet as PC and their TP oxalic acid (OA) and sulfonic acid (ESA) were selected since they are the dominant pesticides applied in autumn in the study area. Interviews with farmers in the catchment conducted in 2008, 2009, 2014, and 2015 further confirm the usage of metazachlor and flufenacet for the main crops in this area (Ulrich et al. 2012, 2018, 2019). Metazachlor is applied at the end of August until the beginning of September to rapeseed, while flufenacet is applied from mid of September until mid/end of October to winter wheat and winter barley. Both herbicides are sprayed either before or right after sprouting. Both PC are stable in water; hence, the main transformation processes take place in the soil by microbiological activity (Laue et al. 1996; Lewis et al. 2016). The environmental fate of the target compounds is related to their physical-chemical properties, and their characteristics are listed in Table 2.

**Table 2:** Physical-chemical properties of the target compounds (*Dechene et al. 2014; Lewis et al. 2016)

Properties	Metazachlor	Flufenacet	Metazachlor sulfonic acid (M-ESA)	Metazachlor oxalic acid (M-OA)	Flufenacet sulfonic acid (F-ESA)	Flufenacet oxalic acid (F-OA)
Water solubility, 20°C	450 mg L^−1^	56 mg L^−1^	*216 g L^−1^	*198 g L^−1^	55 g L^−1^	-
Log P_OW_ (Octanol-water partition coefficient) at pH 7, 20 °C	2.49	3.2	-	-	-	-
Kf_OC_ (organic-carbon sorption coefficient)	53.8–220 ml g^−1^	113–696 ml g^−1^	5 ml g^−1^	24.6 ml g^−1^	12.5 ml g^−1^	14 ml g^−1^
Photolysis in water DT_50_	Stable	Stable	-	-	-	-
Hydrolysis in water DT_50_	Stable	Stable	-	-	-	-
DT_50_ in soil (field experiments)	3–21 d	14–68 d	60–171 d	53–138 d	6–1000 (302) d	6–1000 (11) d
DT_50_ in water phase	216 d	54 d	-	-	-	-
DT_50_ in sediment	21 d	81 d	-	-	-	-
Aquatic invertebrates—acute 48-h EC_50_ *Daphnia magna*	33 mg L^−1^	30.9 mg L^−1^	93.8 mg L^−1^	100 mg L^−1^	>87.3 mg L^−1^	-
Aquatic plants—acute 7-day EC_50_, biomass *Lemna gibba*	0.0162 mg L^−1^	0.002 mg L^−1^	91.9 mg L^−1^	100 mg L^−1^	**>** 79.5 mg L^−1^	> 100 mg L^−1^

- No data available

#### Chemical analyses of target compounds

All samples were left for 24h at 4 °C for sedimentation before the analysis, and the supernatant water was analyzed without any further treatment according to DIN 38407-36:2014-09 by liquid chromatography-mass spectroscopy using an AB Sciex 5500 Qtrap with an injection volume of 100 μL. Chemicals from the companies Merck (methanol LiChrosolv, acetonitril LiChrosolv, ammonium acetate pro analysi, and acidic acid), Fluka (formic acid), and Neochem (standards) were used. The limits of quantification (LOQ) was 0.01 μg L^−1^ for metazachlor and flufenacet and 0.025 μg L^−1^ for the TP in surface water and groundwater. In drinking water, the LOQ of the TP was 0.01 μg L^−1^ for all compounds. Results below the LOQ were set to 0.005 LOQ for illustration in figures and further calculations. The quality criteria of the analyses are listed in Table 3. The repeatability was measured via tenfold injection, the reproducibility according to DIN EN ISO 11352:2013 (DIN 2013), and Koch (2015) including the uncertainty factor *k*=2. The recovery was determined in the sample matrix.

**Table 3:** Quality criteria of the pesticide analyses

Compound	Limit of quantification [μg L^−1^]	Repeatability (relative standard deviation) [%]	Reproducibility [%]	Linearity [μg L^−1^]	Recovery [%]
Metazachlor	0.01	1.5	14.61	0.005-1	100
M-ESA	0.025 0.01 in drinking water	6	23.27	0.005-1	99
M-OA	0.025 0.01 in drinking water	8	26.72	0.005-1	95
Flufenacet	0.01	1.6	19.42	0.005-1	96
F-ESA	0.025 0.01 in drinking water	1.2	16.9	0.005-1	97
F-OA	0.025 0.01 in drinking water	1.2	16.39	0.005-1	107

### Land use and pesticide application data

The land use was mapped through field surveys in spring and early summer in 2016 and 2017 for the vegetation periods 2015/2016 and 2016/2017 with QGIS Version 2.4 (2014) at field resolution (Lei et al. 2019). The vegetation period includes the time from sowing to harvest and lasts from end of August to July/August of the following year.

Farmers of the catchment were interviewed about the following:

- Date of application
- Name of applied product and its concentration in the application solution
- Size of field

From the interviews, information about 26% (2015) and 37% (2016) of the winter grain area in the catchment was gathered. For rapeseed, information about 50% (2015) and 46% (2016) of the area in the catchment were available. This is a high share of information in comparison to other studies where one date of application and the recommended amount of the producers are considered (Fohrer et al. 2007). This data was extrapolated to the mapped total rapeseed/winter grain area in the catchment in the respective years to assess the applied amount of metazachlor and flufenacet at catchment scale and to calculate the export into the Kielstau stream.

## Results and discussion

### Occurrence of TP in selected water body types

**Fig. 2:**
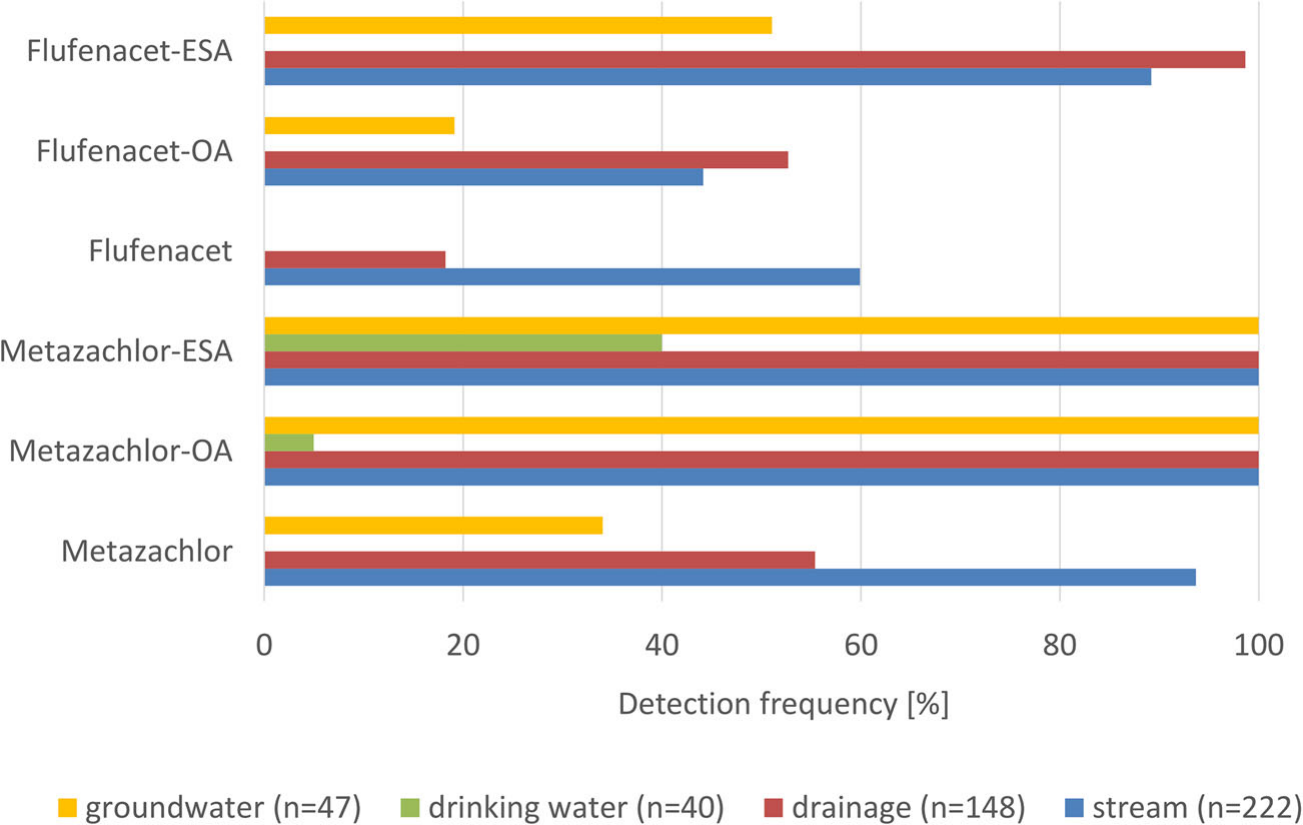
Detection frequencies of target compounds in all water types with *n* = number of samples per water body type, total number of samples = 457

As a first step, the results of the monitoring campaigns were examined in order to observe hot spots of the selected compounds in the different water types. Accordingly, the number of positives were counted and related to the total amount of samples per water type from the entire investigation period (Fig. 2). In all water types, metazachlor and flufenacet were detected in less samples than their TP, except for stream water where the number of flufenacet findings exceeded those of F-OA. Moreover, PC were rarely detected in drinking water or groundwater; only metazachlor was measured in 16 out of 47 samples. Three metazachlor positives were observed in one well and 13 in the second well. PC were mainly detected in stream and drainage water (flufenacet in 160 out of 370 samples; metazachlor in 290 out of 370 samples), while metazachlor-TP were observed in all water types. This also counts for flufenacet-TP, except that they were not detected in drinking water.

Stream and drainage water are fast-reacting water types. In this context, we define fast-reacting as a water body type with no or only a short period of percolation through the soil. Hence, the time for sorption and transformation processes is limited, and the PC can be measured soon after application in these water types. In a study of small water body contamination, similar results were obtained: M-ESA and M-OA were the compounds with the highest detection frequencies (85.5% and 74.5%) in that data set. The PC metazachlor and flufenacet were measured in 10.9% and 6.5% of the samples, while flufenacet-TP were not explicitly mentioned (Wick et al. 2018). The high number of findings, especially levels of metazachlor-TP and F-ESA between 89 and 100% in drainage and stream water, between 51 and 100% in groundwater and partially in drinking water proves their persistence and mobility (Table 2). This highlights the importance of research on their environmental fate and risk assessment.

**Fig. 3:**
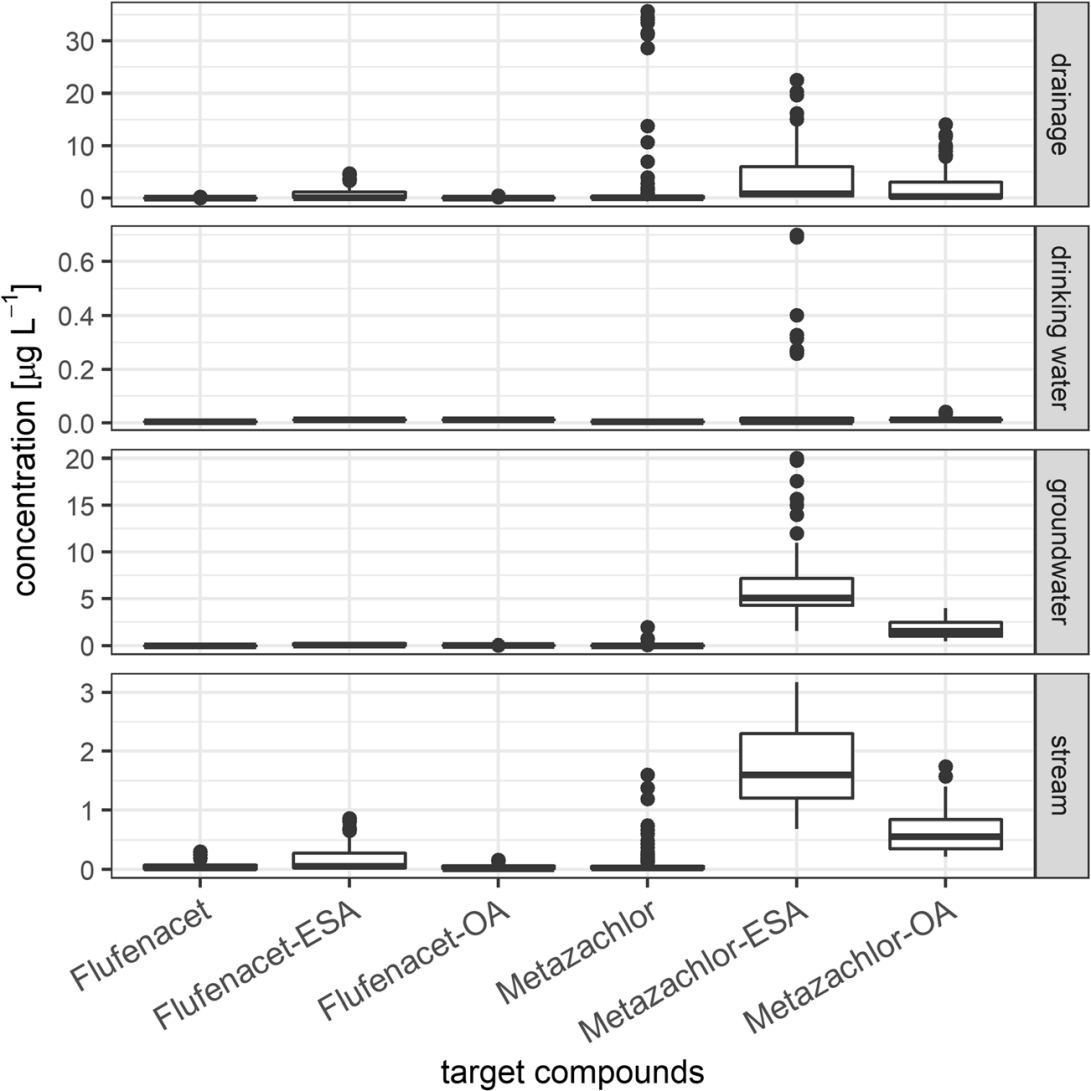
Concentration range of target compounds in all water types from the entire investigation period with boxes, concentration range where 25–75% of the data set are located; dots, outliers of this range; lines, median

Considering the concentrations, the pattern of the six target compounds is similar for all types of water with M-ESA showing the highest concentration followed by M-OA, F-ESA, and F-EOA (Fig. 3). But in single drainage samples, metazachlor was quantified in concentrations up to 35 μg L^−1^. These isolated findings of high metazachlor concentrations were related to an intense rainfall event in September 2016 at the end of the application period (see Chapter 3.3.1). The highest concentrations were detected in the water body types drainage and stream with 35.7 μg metazachlor L-1, 14.1 μg M-OA L-1, 0.30 μg, 22.5 μg M-ESA L-1, flufenacet L-1, 0.45 μg F-OA L-1, and 4.67 μg F-ESA L-1. The transport of water and dissolved pesticides to these water types is fast. The potential for sorption or microbiological transformation is limited for drainage water due to the short soil passage to depth of ca. 60–120 cm where drainage pipes are usually located (Kreuger 1998; Willkommen et al. 2019; Ulrich et al. 2019). Pathways such as surface runoff, drainage water, and interflow, all of which contribute to the stream flow, have a lower potential for transformation and retention compared to the potential of percolation to greater depth of groundwater and drinking water sources (Lange et al. 2018). The results gained in this study are in accordance with the findings of other studies indicating higher detection frequencies and concentrations of TP than of the corresponding PC (Kolpin et al. 2004; Wick et al. 2018; Kiefer et al. 2019). The results reveal that fast-reacting water bodies like drainage and stream water are prone to PC and TP contamination while groundwater and drinking water sources as slow-reacting water types which are mainly affected by TP but to a lesser extent than drainage and stream water.

### Effect of distinct hydrological conditions on the export of TP

The hydrological conditions of the sampling campaigns varied significantly due to different amounts of precipitation and its pattern which impacted the release of target compounds to the drainage system and to the stream.

#### Precipitation patterns and pesticide application

The common period when stream water was sampled in both years was from 25 August to 29 November with precipitation sums of 560 mm in 2015 and 388 mm in 2016 (Table 4). 2015 was characterized by precipitation events at the beginning of October and continuous precipitation from beginning of November to the end of the sampling. In 2016, a small-scale rainfall event occurred on 4 September which accounted for 28 to 90 mm according to the location in the catchment. In the middle of October, a rainy period lasting 5 days was recorded.

**Table 4:** Characteristics of precipitation during sampling 2015 and 2016

	Monthly sum 2015 [mm]	Monthly sum 2016 [mm]	Rainy days 2015	Rainy days 2016
August	91	66	12	17
September	83	93	19	8
October	76	104	11	15
November	165	63	21	14
December	145	62	22	14
Sum	560	388	85	68

The results of the land use mapping at catchment scale and the application data resulting from the interviews of the farmers are shown in Table 5. Metazachlor and flufenacet have been approved in Germany around 1982 and 1998 (BBA 1982) and have been applied in the study area at least since 2008 (Ulrich et al. 2012). Metazachlor was applied in amounts of 0.082–1 kg/ha and flufenacet in amounts of 0.042–0.24 kg/ha, respectively. In the catchment, the share of rapeseed area differed from 11 to 14% and that of flufenacet from 33 to 41%, respectively, in the time span 2008–2016 (Ulrich et al. 2012; Lei et al. 2019).

**Table 5:** Area and pesticide application data of rapeseed and winter grain fields at catchment scale in 2015 and 2016 with application amounts calculated for the active compounds metazachlor and flufenacet

	Area 2015 [ha]	Applied pesticide 2015 [kg]	Mean application 2015 [kg/ha]	Area 2016 [ha]	Applied pesticide 2016 [kg]	Mean application 2016 [kg/ha]
Metazachlor (rapeseed)	525	264	0.5	559	278	0.5
Flufenacet (winter grain)	2012	228	0.11	1592	227	0.14

**Fig. 4:**
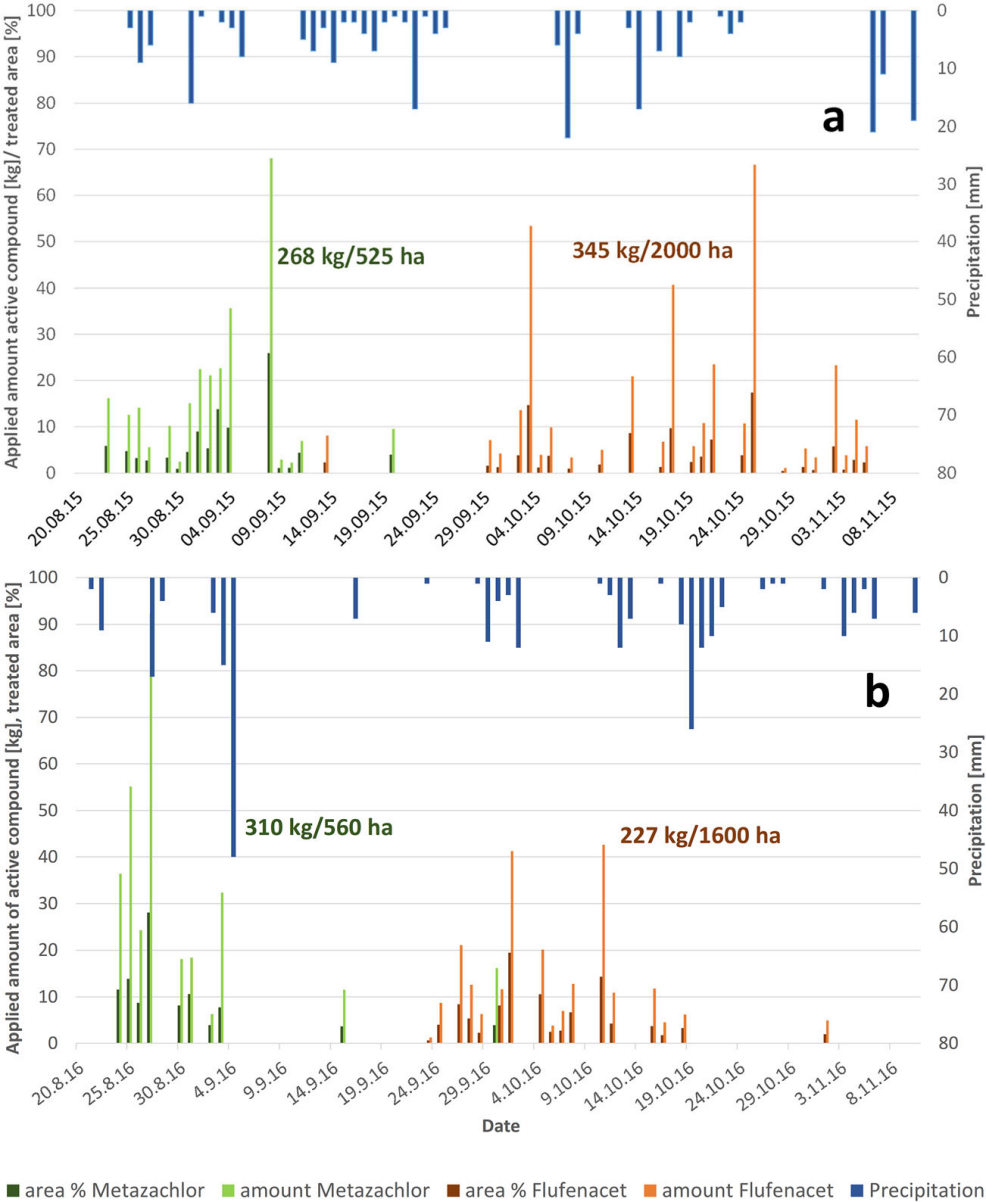
Application dates and amounts (daily sums) from interviews with farmers of the catchment as well as precipitation (daily sums) in 2015 (**a**) and 2016 (**b**) in the catchment with total amount and total area of crop in the entire catchment

The combined graphic of application dates/amounts and precipitation demonstrates that in 2015, one rainfall event on 27 August 2015 occurred in the application period of metazachlor, while several events higher than 5mm/d took place in the flufenacet application period (Fig. 4). In 2016, the 100-year rainfall on 4 September was recorded almost at the end of the metazachlor application period. Hence, most fields were treated, and high metazachlor export could be expected. Subsequently, several smaller events occurred during the flufenacet application of which the rainy period from 18 October to 23 October 2016 at the end of the applications had the highest effect on the export of flufenacet compounds (see Chapter 2.23). The distribution of precipitation within a catchment is a crucial factor. Although the study area is a lowland and has a relatively small size of 50 km^2^, the variability of precipitation has to be considered when data are recorded from only one climate station. Especially the rainfall event on 4 September 2016 showed values of 26 mm, 45 mm, and 90 mm according to our climate station and reports of farmers within the catchment and depending on the location.

#### Tile drainage

**Fig. 5:**
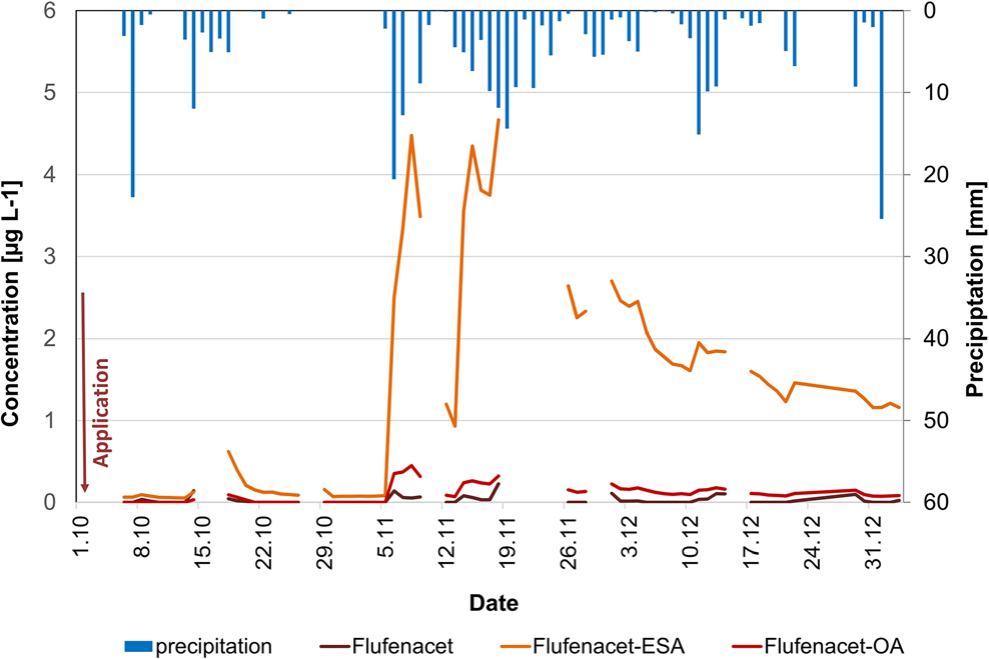
Concentrations of flufenacet and its TP in tile drainage water in 2015 and daily precipitation sums, application of flufenacet on 1 October 2015

In 2015, flufenacet was applied to this field on 1 October 2015 with 140 g/ha. A slight increase of TP concentrations was measured after the first rainfall on 15 October 2015 (Fig. 5). Approximately 4 weeks after application, the concentrations of F-ESA and F-OA rose to 4.35 μg L^−1^ and 0.448 μg L^−1^ after a rainfall event and decreased constantly until the end of the campaign. The maximum height of the peak cannot be reported since some data is missing for this period. In an incubation experiment with distinct amended soils and temperatures, F-ESA and F-OA were detected in maximum concentrations 32 to 41 days after flufenacet application. This reveals that a low sorption potential to soil and higher temperatures can increase the transformation for flufenacet (Marín-Benito et al. 2019). During the entire sampling period, F-ESA concentrations were highest (4.67 μg L^−1^), followed by F-OA (0.448 μg L^−1^), and the PC flufenacet (0.333 μg L^−1^). Before application, F-ESA concentrations were 0.064 μg L^−1^ likely as residues from the previous year application. But also, M-ESA and M–OA were detected at levels up to 0.820 and 0.107 μg L^−1^ (not shown), respectively, though the corresponding PC was applied last in autumn 2013.

**Fig. 6:**
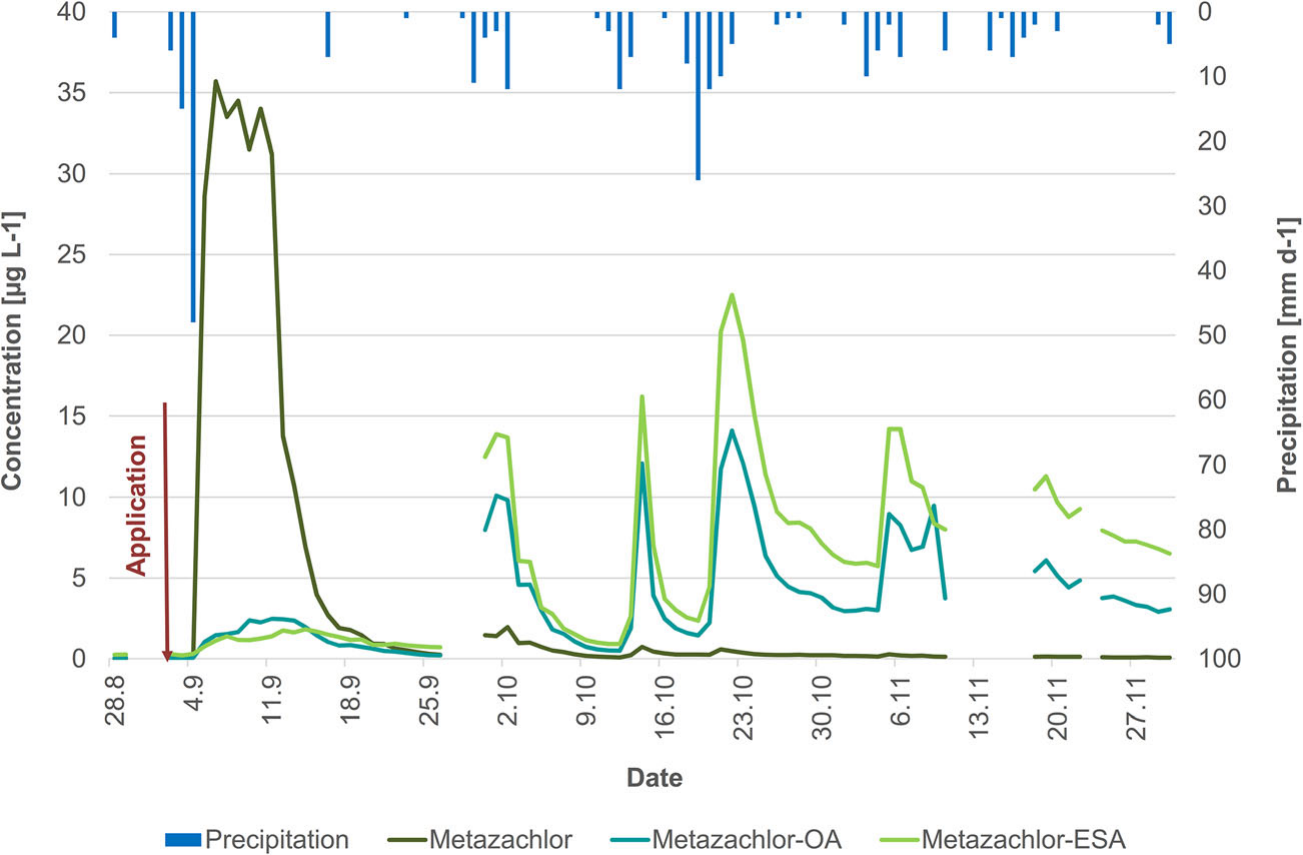
Concentrations of metazachlor and its TP in tile drainage water in 2016 and daily precipitation sums, application of metazachlor on 3 September 2016

In 2016, metazachlor was applied to this field on 3 September 2016 with 750 g/ha to the tile drained field. As previously stated, the rainfall event on 04 September 2016 differed widely within the catchment, with up to 90 mm being reported by the owner of the drained field. It caused extreme surface runoff from the field and high discharges from the drainage pipe, hence severe flooding. Metazachlor concentrations in the following days were up to 35 μg L^−1^, which decreased fast but still showed levels of 0.074 μg L^−1^ at the end of the sampling 89 days after application (Fig. 6). The TP concentrations after this rainfall event were 2.48 μg M-OA L^−1^ and 1.62 M-ESA μg L^−1^. These can be assumed as residues in the soil resulting from former applications since the transformation of metazachlor needs more time to form and release high TP concentrations (Allen and Walker 1988; Baćmaga et al. 2014; Lewis et al. 2016). After approximately 4 weeks, the TP were released after a dry period triggered by rainfall. Maximum concentrations of 22.5 μg M-ESA L^−1^ and 14.1 μg M-OA L^−1^ were measured 7 weeks after application. These decreased continuously until the end of the sampling, and except for the high metazachlor export at the beginning of September, the order of concentration height was again M-ESA > M-OA > metazachlor. Before the current application, 0.267 μg M-ESA L^−1^ and 0.070 μg M-OA L^−1^, likely as residues from the 2013 application, were detected as well as F-ESA with 0.148 μg L^−1^ (not shown) during the extreme rainfall event as a residue from the previous year’s application.

In both years and despite the varying hydrological conditions and different compounds, the pattern of TP export was similar: after approximately 4 weeks, the concentration of the TP increased considerably revealing that the transformation process in soil needs some time until the TP are released and found in water samples. In general, the concentrations of the metazachlor compounds were higher than those of the flufenacet group. The higher application amount per hectare of metazachlor (750 g/ha) compared to flufenacet (140 g/ha) can be considered as one factor contributing to this. The more likely scenario is that higher mobility of metazachlor and its TP rather than that of flufenacet and its TP leads to higher concentrations, especially during rainfall-induced discharge peaks. These high pulses put a risk to aquatic organisms when contaminated drainage water enters receiving waters as it was previously observed in a mesocosm study for metazachlor by Mohr et al. (2007, 2008). Hence, pre-cleaning of drainage water with end-of-pipe solutions has to be discussed to improve the water quality.

#### Stream water

##### Metazachlor and its TP

**Fig. 7:**
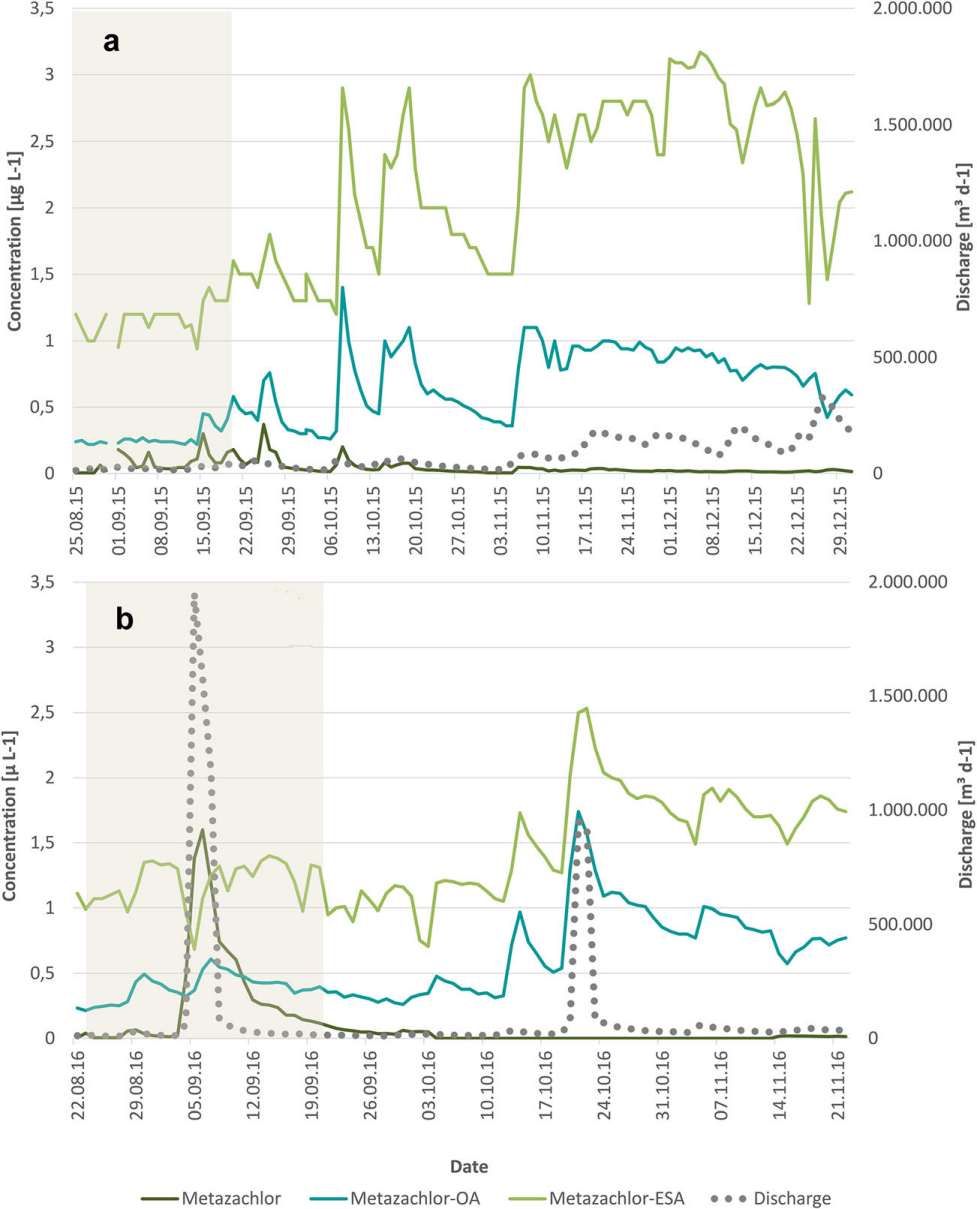
Concentrations of metazachlor and its TP M-ESA and M-OA at the outlet of the catchment in the monitoring periods of 2015 (**a**) and 2016 (**b**); gray area, application period

In 2015, daily metazachlor concentrations varied from <LOQ to 0.34 μg L^−1^ with the maximum 25 September 2015 (Fig. 7). Due to its short half-life time, concentrations decreased fast and fell below 0.02 μg L^−1^ from mid-November. Metazachlor-TP were already detected as residues before the start of the application period in concentrations of 0.24 μg M-OA L^−1^ and 1.6 μg M-ESA L^−1^. Residues from the previous year application of metazachlor were also reported in pond and drainage water (Ulrich et al. 2018, 2019). The concentrations of the TP followed the PC pattern solely differing in their values which were on average 30 times (M-OA) and 100 times (M-ESA) higher than the PC’s. In the second half of the sampling, this rate increased due to the fast dissipation of metazachlor and a partial increase of TP concentrations up to 82 to 100 times. The first slight increase of TP concentrations after application was observed 2 weeks later and increased considerably at the end of September, approximately 4 weeks after application of the PC. According to Allen and Walker (1988) and Lewis et al. (2016), TP are mainly formed in the soil but can be detected in photolytic lab experiments as well (Mantzos et al. 2017; Hensen et al. 2019). Hence, microbiological and photolytic processes transform metazachlor into these TP. Maximum daily loads of 182 g M-OA and 637 g M-ESA occurred on 25–26 December 2015 and were mainly caused by higher discharge levels rather than higher concentration levels. The highest concentrations of 1.4 μg M-OA L^−1^ and 3.17 μg M-ESA L^−1^ were detected on 8 October and 6 December 2015, respectively.

Referring to the extreme rainfall event on 4 September 2016, maximum metazachlor concentrations and loads were observed on 5 September 2016 with 1.6 μg L^−1^ and 2695 g. The second highest load of 56 g occurred on 21 October 2015, when concentrations (0.066 μg L^−1^) and discharge peaked a second time. The values of both events emphasize the extremely high export of metazachlor with an almost 160 times higher maximum daily load at the beginning of September 2016 than in 2015. Similar to 2015, residues of metazachlor-TP were detected before the current application period in concentrations of 0.232 μg M-OA L^−1^ and 1.11 μg M-ESA L^−1^. Again, the TP concentrations followed the PC pattern, and concentrations first increased considerably at the beginning of October. Maximum concentrations and loads were detected on 21 October 2016 and 22 October 2016 due to a 3-day rainy period of 32 mm. The findings amounted to 1.74 μg M-OA L^−1^ and 2.53 μg M-ESA L^−1^, while highest daily loads were 20.8 g M-OA and 30.3 g M-ESA.

Comparing the exported loads in the same periods of 2015 and 2016 (Table 6) at the outlet of the catchment, metazachlor loads were considerably higher in 2016 than in 2015 and vice versa for the TP.

**Table 6:** Exported loads of metazachlor and its TP from 24 August to 6 December in the years 2015 and 2016 in stream water

Year	∑ Metazachlor [g]	∑ Metazachlor [%]	∑ M-OA [g]	∑ M-ESA [g]	∑ total M [g]	∑ total M [%]
2015	225	0.09	2780	8568	11,573	4.38
2016	6991	2.51	663	1581	9235	3.32

While the export of metazachlor in 2015 is in the same range as the study of Kreuger (1998) with 0.32–0.44%, the value of 2016 is in the upper range of general pesticide export of 1–5% of the applied amount due to the extreme rainfall event on 4 September 2016 (Imfeld et al. 2013; Gassmann et al. 2015). Due to the high metazachlor export, less metazachlor was available for transformation processes. Hence, exported metazachlor-TP loads in 2016 were 4 to 5 times lower than 2015. Moreover, the high TP loads in 2015 were caused by the rainy period from mid-November onwards, but in 2016, November and December were dry and reduced the potential for TP release. The area of rapeseed was similar in both years and did not affect the losses. Taking into account that one molecule of metazachlor can be transformed into one TP, the TP loads need to be included into the balance. Thus, the total export of metazachlor and TP was higher in 2015 since the higher TP loads in 2015 offset the extreme metazachlor load in 2016. To our knowledge, the inclusion of pesticide-TP into the export balance has been carried out in our study for the first time; therefore, comparisons to other studies cannot be drawn.

##### Flufenacet and its TP

**Fig. 8:**
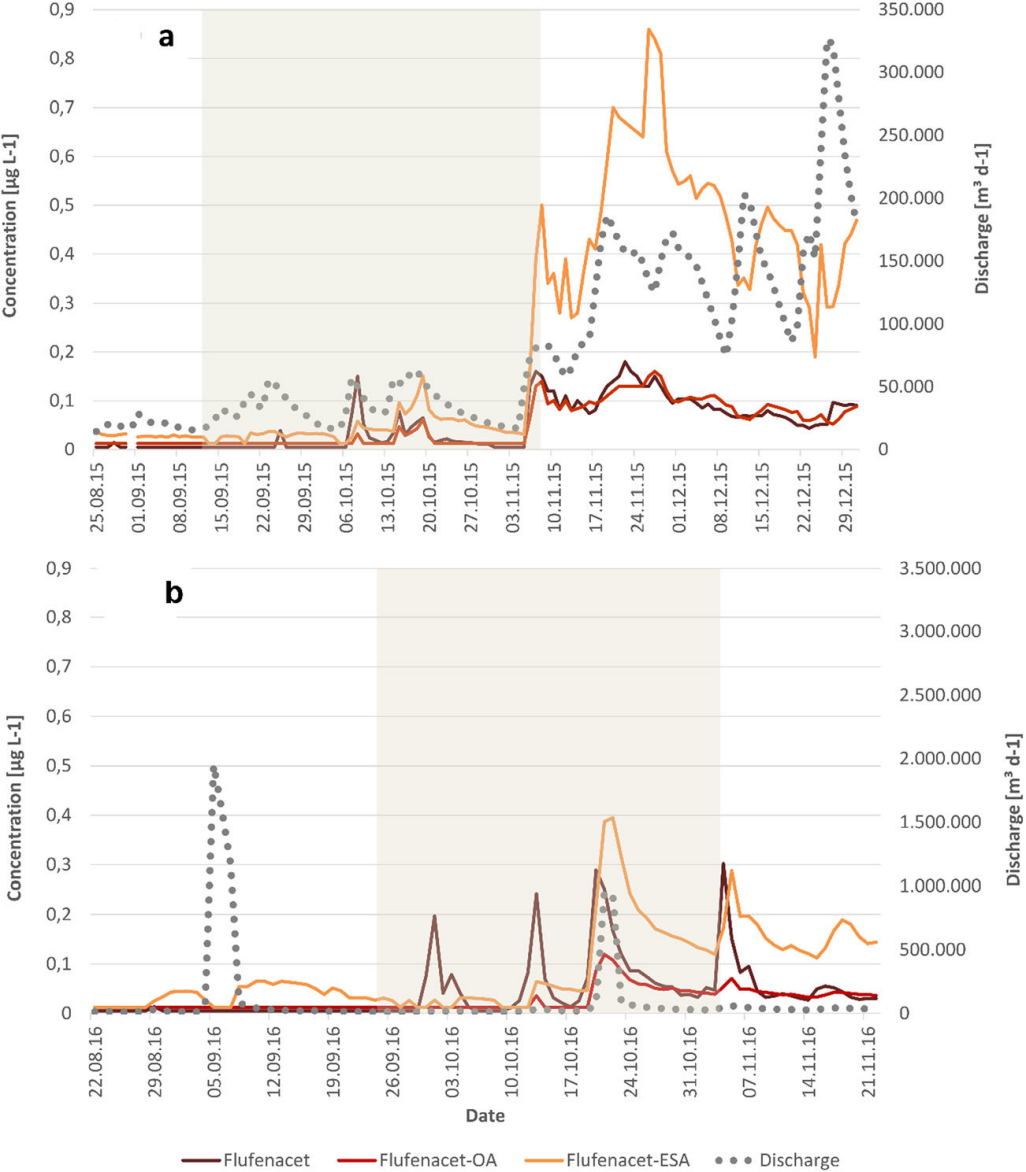
Concentrations of flufenacet and its TP F-ESA and F-OA at the outlet of the catchment in the monitoring periods of 2015 (**a**) and 2016 (**b**); gray area, application period

For flufenacet in 2015, daily concentrations were in the range of <LOQ to 0.18 μg L^−1^ in the rainy period from 19 November to 24 November 2015 (Fig. 8). The first flufenacet peak in the application period of 0.15 μg L^−1^ was detected 2 days after a 23-mm rainfall event on 6 October 2015, which followed 10 dry days. Because the main application was in October, the export of flufenacet grew over time and increased again in the intense rainfall period from the beginning of November due to high discharges. While F-OA concentrations were below the LOQ prior to the application period, F-ESA residues were measured in concentrations up to 0.36 μg L^−1^. The first slight TP peaks occurred at the beginning of October after a quarter to a third of the fields in the catchment were sprayed, while considerable TP concentrations started on 8 November 2015 following 2 days of 21- and 11-mm rainfall. This was approximately 4 weeks after a relevant share of winter grain fields were treated and at the end of the application period. Maximum daily TP concentrations were measured in the same period as those of the PC with 0.16 μg F-OA L^−1^ and 0.86 μg F-ESA L^−1^. Similar to the pattern of flufenacet, TP concentrations and loads increased slightly in October and extremely with the intense rainfall period from the beginning of November onwards due to high discharge. The last and highest discharge peak of the sampling period at the end of December did not cause very high exports of flufenacet and its TP. It is assumed that a subsequent delivery of the target compounds from the soil storage was limited because most of it had already been washed out.

In 2016, the daily concentrations amounted from <LOQ to 0.302 flufenacet μg L^−1^ g with the maximum on 4 November 2016 following a 4-day rainy period of 22-mm rainfall and accompanied by high discharge. The first flufenacet peak occurred on 1 October 2016 with concentrations of 0.197 μg L^−1^ but still during low discharge. None of the flufenacet compounds were detected prior to the application period. First slight TP peaks were observed in mid-October, but concentrations and loads were marginal until 20 October 2016, when several days of rainfall increased TP concentrations and discharge to maximum values of 0.119 μg F-OA L^−1^ and 0.395 μg F-ESA L^−1^. Following rainfall events were below 5 mm d^−1^, and no relevant TP or PC loads were measured until the end of the sampling.

The loss rate of flufenacet and its TP was considerably lower in 2016 than in 2015 (Table 7). The factor varies from 6.8 for flufenacet to 9.0 and 9.9 for the respective TP.

**Table 7:** Exported loads of flufenacet and its TP from 24 August to 6 December in the years 2015 and 2016 in stream water

Year	∑ Flufenacet [g]	∑ Flufenacet [%]	∑ F-OA [g]	∑ F-ESA [g]	∑ total F [g]	∑ total F [%]
2015	320	0.14	272	975	1567	0.69
2016	47	0.02	30	98	175	0.08

The percentage loss of flufenacet of 0.02 % is in the same range as observed in this catchment in 2009 (Ulrich et al. 2012) and in the study of Willkommen et al. (2019). Mainly the continuous rainfall events from the beginning of November onwards in 2015 generated constantly high export rates of flufenacet and its TP. In 2016, single rainfall events led to selective losses which were 10 times lower for flufenacet and up to 20 times lower for the TP when compared to 2015. Relevant rainfall events occurred outside the application period, thereby producing less of an export than in 2015. The area of winter grain decreased from 2012 to 1592 ha, but at the same time, the applied flufenacet amount increased from 0.11 to 0.14 g/ha; hence, the applied amount at catchment scale was comparable. Again, comparable loss rates for all flufenacet compounds including TP have not been reported yet.

For the assessment of the data, according to the Environmental Quality Standards (EQS), acute doses must be applied according to the sampling scheme. In 2015, neither flufenacet nor metazachlor exceeded the EQS of 0.200 μg L^−1^ (OGewV 2016) and 1.25 μg L^−1^ of the Regulatory Accepted Concentration (RAC) (UBA 2020a). However, in 2016, the flufenacet threshold was exceeded four times. In previous studies sampling small and medium size streams, flufenacet exceeded the EQS acute in 1–1.2% of all samples (Brinke et al. 2016), which is in the same range found in our study (1.8%). For metazachlor, the RAC was exceeded in two samples and almost in a third one, which is equal to 0.9% of the stream samples. This is comparable to the findings of Brinke et al. (2016) of ca. 0.7%. Environmental Quality Standards for TP in surface water have not been considered so far, hereby hampering an integrated ecotoxicological assessment of water to evaluate the potential threat of aquatic organisms. The acute EC_50_ values of the TP for aquatic invertebrates and algae are in the range of >30 mg L^−1^ for the target compounds. The acute EC_50_ values for algae were only 16.2 μg L^−1^ for metazachlor and 2 μg L^−1^ for flufenacet and were not exceeded by any measurement within this study. Besides direct effects on aquatic life, indirect impacts by changes of the habitat structure need to be considered as well (Mohr et al. 2007, 2008). Hence, suitable ecotoxicological studies should be carried out as an integrated approach to ensure a minimum acceptable threat to aquatic organisms, considering that our study reveals a long-term exposition. In a database, collecting pesticide and TP concentrations from 2005 to 2015, the TP M-ESA and M-OA were the compounds with the highest detection frequency, the PC metazachlor, and flufenacet followed at 28th and 42nd position out of 82 compounds (Wick et al. 2018). These data reveal the omnipresent distribution of TP and their high relevance.

### Vulnerability of drinking water and groundwater to TP leaching

#### Shallow groundwater

The highest concentrations were found for metazachlor-ESA at both groundwater wells with up to 20 μg L^−1^ (Table 8). Also, minimum values at both sites were below 5 μg L^−1^ (GW1: 2.57 μg L^−1^; GW2: 1.5 μg L^−1^). Furthermore, M-OA was found at higher concentrations than metazachlor. The PC metazachlor itself was detected in GW2 after an extreme rainfall right after application.

**Table 8:** Concentrations of target compounds in shallow groundwater wells; *LOQ* limit of quantification

Parameter	Groundwater well	Number of samples	Median [μg L^−1^]	Min [μg L^−1^]	Max [μg L^−1^]
Metazachlor	GW1	23	<LOQ	<LOQ	0.03
Metazachlor-ESA			7.41	2.57	20.00
Metazachlor-OA			1.30	0.55	2.98
Flufenacet			<LOQ	<LOQ	<LOQ
Flufenacet-ESA			<LOQ	<LOQ	<LOQ
Flufenacet-OA			<LOQ	<LOQ	<LOQ
Metazachlor	GW2	24	0.02	<LOQ	1.96
Metazachlor-ESA			4.58	1.50	6.90
Metazachlor-OA			2.05	0.47	3.99
Flufenacet			<LOQ	<LOQ	<LOQ
Flufenacet-ESA			0.25	0.07	0.38
Flufenacet-OA			<LOQ	<LOQ	0.04

Conversely, the risk of leaching for flufenacet and its TP seems to be much lower since only low concentrations were detected at GW2. The absence of flufenacet and its TP in GW 1 cannot be valued, since there was no application of flufenacet near GW1 during our studies.

GW1 is expected to be at higher risk for contamination because there is no cover layer in the sandy soil. Consequently, mobile compounds like TP of metazachlor can be transported more easily by seepage into groundwater. This is confirmed by the high concentrations of up to 20 μg M-ESA L^−1^ and 2.98 M-OA L^−1^ as well as relevant concentrations of up to 0.03 μg metazachlor L^−1^. The profile of GW2 suggests better protection against pollution of TP due to silty layers in the soil column. However, there are high groundwater levels, and the filter strip starts at 1.50 below surface fostering leaching of substances into the groundwater. The silty layer might be too thin to prevent leaching of PC and their TP.

In summary, especially metazachlor and its TP were observed in both groundwater wells. For shallow groundwater, our results indicate at least the risk of exceeding thresholds of the German groundwater directive of 0.1 μg L^−1^ for PC, here metazachlor. Since non-relevant metabolites/TP like those in our study are not considered by the German Groundwater Directive and the European Drinking Water Directive (98/83/EC 1998), we used the health-related threshold value GOW (Gesundheitlicher Orientierungswert) established by the German Environmental Protection Agency (UBA 2020a). The GOW is derived for compounds without a limit value in the European Drinking Water Directive (98/83/EC 1998) such as most TP and which occur in drinking water at relevant levels. The GOW threshold values are intended for drinking water hygiene purposes; they are also applied to groundwater. Regarding the metazachlor-TP, the defined GOW of 3 μg L^−1^ was temporarily exceeded. This finding is in line with Reemtsma et al. (2013b), who set both metabolites of metazachlor as prominent substances in groundwater. Our findings and the results of Reemtsma et al. (2013b) indicate potential future risks, if these compounds are transported to deeper aquifers which are used for drinking water.

#### Drinking water

The depth of the five drinking water wells (DW1-DW5) varied in the range of 17 to 100 m (Table 9). The PC metazachlor and flufenacet were not detected in any well. Furthermore, the wells with a depth larger than 48 m (wells 2, 3, 5) did not show any positives of any TP. In DW 4, M-ESA was constantly observed at levels slightly above the LOQ of 0.01 μg L^−1^. In DW 1, M-OA was measured in three of the eight samples with concentrations up to 0.041 μg L^−1^. M-ESA was detected in all samples of DW 1, starting with the highest concentration of 0.7 μg L^−1^ at the beginning of the monitoring in November 2015 and March 2016 and decreasing to 0.26–0.032 μg L^−1^ from November 2016 until the end of the campaign (Table 9).

**Table 9:** Target compound concentrations in drinking water wells during the sampling from November 2015 to September 2017; *LOQ* limit of quantification

Parameter/well	DW1 [μg L^−1^]	DW2 [μg L^−1^]	DW3 [μg L^−1^]	DW4 [μg L^−1^]	DW5 [μg L^−1^]
Depth of well [m]	17	100	99	48	57
Metazachlor	<LOQ	<LOQ	<LOQ	<LOQ	<LOQ
Metazachlor-OA	<LOQ-0.041	<LOQ	<LOQ	<LOQ	<LOQ
Metazachlor-ESA	0.266–0.700	<LOQ	<LOQ	0.012–0.019	<LOQ
Flufenacet	<LOQ	<LOQ	<LOQ	<LOQ	<LOQ
Flufenacet-OA	<LOQ	<LOQ	<LOQ	<LOQ	<LOQ
Flufenacet-ESA	<LOQ	<LOQ	<LOQ	<LOQ	<LOQ

The results show that drinking water wells with a larger depth seem to be more protected against leaching than shallower ones. The soil profiles reveal several layers that prevent fast transport of water and dissolved compounds into deeper layers and into groundwater. Well DW4, which shows consistent values for M-ESA above the LOD, is installed in sandy soils with clay layers from 7–10 m to 30–35 m. Contrary to the permanent and uniform concentrations in DW4, the M-ESA concentrations in DW1 show temporal dynamics. In general, dynamics within quarterly samples are not expected at depth of 17 m. With a 50-m distance to DW1 and being separated by a road, a neighboring field was used for growing rapeseed, to which metazachlor could have been applied in autumn 2014. Transport of the TP resulting from this field can be considered since it is in the catchment of the well. Due to its high mobility and persistence, M-ESA was detected in studies of Reemtsma et al. (2013a) and Ulrich et al. (2018) up to 3 years after application of the PC metazachlor in surface and drainage water. Moreover, it has to be considered that DW1 was not properly sealed during the construction, and percolation along the well occurred. A second explanation could be that layers with low permeability have clearance holes from former wells for drinking water or irrigation purposes through which pesticides leach downwards. Since DW1 was installed in the 1960s, soil profiles are not available, and further conclusions about the findings cannot be drawn. Kolychalow et al. (2012) conducted a similar study in private drinking water wells. Their results demonstrate considerably higher concentrations and detection frequencies of TP (of atrazine, chloridazone, dichlofluanide, tolylfluanide) than their PC. Similar to our results, concentrations and detection frequencies decreased with increasing depth of well. Our findings show that TP but not the corresponding PC are able to reach groundwater used for drinking water purposes according to their mobility and persistence. Contrary to our results, Quintana et al. (2019) detected the PC metazachlor in drinking water of Barcelona’s metropolitan area with 0.012 μg L^−1^; TP were not analyzed.

To assess the risk of the metazachlor-TP concentrations, they are compared to GOW limits. The GOW of both metazachlor-TP are 3 μg L^−1^ and 1 μg L^−1^ for F-ESA/F-OA (UBA 2020b), which is clearly above the concentration we found in the drinking water wells. Thus, a risk for human health is not yet expected. Nevertheless, many TP can hardly be removed from the water cycle, and some of them have the potential to be transformed into toxicologically relevant TP during drinking water processing. Hence, TP are considered as a potential risk to drinking water in the long run (Kümmerer 2010; Dieter 2010).

The slow-reacting compartments already show a vulnerability to leaching compounds. M-ESA with highest mobility and persistence of the selected target compounds was detected in both groundwater and drinking water wells. Due to the depth, concentrations in drinking water were lower than in shallow groundwater. The longer soil passage and more/thicker sealing layers have a protecting effect on these water types compared to drainage and stream water. For shallow groundwater, the field management and the resulting pesticide applications are another crucial factor. Nevertheless, the detection of pesticide-TP in drinking water wells should be a warning signal.

In summary, our study exhibits the omnipresent detection of TP in all types of water within a catchment. The following patterns were observed:

- Due to their increased mobility, TP are detected more frequently, consistently, and in higher concentrations than the corresponding PC. Metazachlor-TP findings are more relevant in terms of concentration and detection frequency than flufenacet-TP.
- The higher TP-persistence seems to cause findings up to 1 or more years after PC application.
- In tile drainage and surface water, low TP-concentrations were detected approximately 2 weeks after application (drainage), or after a main share of fields were sprayed (catchment scale), substantial TP-concentrations were detected approximately 4 weeks later.

Rainfall patterns considerably affect the formation of TP and their export to fast-reacting compartments. Though the volume of the rainfall event(s) is important to initiate transport, the time between rain and application considerably determines whether or not PC, TP, or both are relocated. Compared to surface water, the ground and drinking water are more protected against pesticide pollution by the soil layers above. Thus, sorption, degradation, and dilution processes take place during the soil passage and decrease the potential for contamination of ground and drinking water. Nevertheless, especially the mobile and persistent TP were detected in both types of water revealing that contaminants may reach deep aquifers as well.

## Conclusion

The main focus of our study was to demonstrate whether TP are limited to certain water body types or whether they are widely spread. The integrated assessment approach of our study reveals the omnipresence of TP in relevant water bodies. Since a rapid degradation of modern pesticides to TP is intended to avoid persistence and bioaccumulation of the PC, it is not surprising that TP are now detected frequently in high concentrations and frequencies. Due to the TP-persistence, aquatic organisms can be harmed not only by typical peaks after application but also by chronic exposure. So far, there is a gap of information about TP properties, and (eco-)toxicities and environmental quality standards are not defined for surface water. Both aspects hamper an overall risk assessment of an applied PC. The TP findings in groundwater and private drinking water wells indicate the vulnerability of slow-reacting water bodies to leaching contaminants. Over time, more TP can be expected to leach to groundwater used for drinking water and accumulate there. Hence, drinking water suppliers may have to face drinking water treatment to remove TP in the future. Measures for the protection of ground and drinking water are mainly based on improved management of pesticide application schemes. Besides, regulations need to be adapted to the current stage of knowledge. In general, the gap of the environmental fate of TP has to be closed by consequent monitoring of these compounds and intense research on specific behavior and (eco)-toxicology. Society, regulators, researchers, and producers have to agree to which level contamination of drinking water will be accepted, e.g., whether the standards of the Drinking Water Directive of 0.1/0.5 μg L^−1^ should be applied to non-relevant metabolites as well. In any case, the monitoring of TP needs to be integrated into a regular state-of-the-art water quality monitoring program.

## Data Availability

The data sets used and/or analyzed during the current study are available from the corresponding author on request.
